# Supplementary material for: A new method of off-site inverse carbon accounting and its application in agriculture carbon measurement
Source: PLoS One. 2026 Feb 9;21(2):e0334270. doi: 10.1371/journal.pone.0334270 (PMC12885257; doi:10.1371/journal.pone.0334270)
Supplement: Attachment — Submitted filename: reply ro reviewers - PONE-D-25-52174.docx [file pone.0334270.s004.docx]

We feel really grateful for the reviewer’s suggestions and commends. Some perspectives have greatly enriched and deepened my academic understanding in the topic and area. It benefits us more than this paper itself. Besides answering all the questions, we have greatly improved this article. The following are the responds to the comments and questions.

**For reviewer 1:**

1. The description of the LSTM model is cursory. Key details are missing, such as: the network architecture (number of layers, neurons), hyperparameters (learning rate, optimizer, number of epochs), the specific input features (beyond "trajectories"), the training/validation split, and the loss function used. The statement that the LSTM "learned patterns and generated numerous sample paths" is not a substitute for a rigorous description.

【Reply】 Thank you for highlighting this issue. In the revised version, we provide a comprehensive LSTM description with all requested technical details. We also add an additional paragraph that explicitly specifies the network architecture (layers and hidden units), input formulation (trajectories with 20-second sliding windows predicting t+1), data preprocessing (mapminmax scaling to [0,1]), training protocol (chronological 80/20 split, no shuffling), optimization settings (Adam, initial learning rate 0.01, mini-batch size 32, up to 100 epochs, piecewise schedule with a 0.1 drop after 300 epochs), and the loss function (MSE), along with evaluation metrics (RMSE, MAE, R²) and the recursive multi-step forecasting procedure (90-second horizon). This replaces the previous cursory phrasing and ensures a rigorous, reproducible specification. The new paragraph is located at the same place under discussion in the Section “Analysis on real case of burning straw”:

“To model and forecast these trajectories, we employed a univariate LSTM regression model implemented in MATLAB (Deep Learning Toolbox). The input to the network consisted of sliding windows of length 20 seconds (history = 20) taken from the trajectory; for each window, the target was the subsequent value at time t+1. Before training, both inputs and targets were linearly scaled to [0, 1] using mapminmax, with parameters stored for inverse transformation. We split the dataset chronologically into training (80\%) and test (20\%) sets without shuffling to preserve temporal order. The network architecture comprised: a sequence input layer (dimension = 20), a single LSTM layer with 4 hidden units and OutputMode = last, a ReLU activation layer, and a fully connected layer with a single neuron, followed by a regression output layer. The model was trained using the Adam optimizer with an initial learning rate of 0.01, a piecewise learning-rate schedule (drop factor 0.1 every 300 epochs), a mini-batch size of 32, and a maximum of 100 epochs. The loss function was mean squared error (MSE) computed on the training mini-batches; performance was further summarized using RMSE, MAE, and R-squared on both the training and held-out test sets. After training, we generated out-of-sample forecasts recursively: the model predicted one step ahead, and the prediction was fed back with the most recent 19 observed/predicted points to form the next 20-point input window. We produced 90-second ahead forecasts per sequence and reported both raw inverse-transformed outputs and an amplitude-normalized variant used for visualization. Figure~\ref{fig:lstmgraphs} presents the training history, in-sample and out-of-sample fits, prediction–truth scatter plots, and multi-step forecasts, illustrating the LSTM learning and prediction workflow.”

2. Table 2 is a good summary but lacks a direct, quantitative comparison of the authors' method against any of the listed alternatives (e.g., a simple Gaussian plume model) using the same dataset. Claims of superiority in accuracy and cost-effectiveness remain qualitative.

【Reply】Thank you for this constructive suggestion. We agree that a head-to-head quantitative benchmark (e.g., against a Gaussian plume/puff model) on the same dataset would be informative; however, such models require inputs that were intentionally not collected in our low-cost field design—namely high-resolution, time-resolved meteorology (stability class, boundary-layer height, vertical temperature gradients), terrain and roughness maps, plume rise/heat release rates, chemistry or deposition parameters, and often multi-sensor arrays for calibration/validation. By contrast, our method operates with minimal information: a short smoke test to obtain local trajectory snippets and basic wind direction/speed, then uses LSTM-driven short-horizon path forecasts to parameterize a Brownian framework for closed-form inversion. This asymmetry in data requirements makes strict quantitative comparisons difficult and potentially biased: when fed rich inputs, Gaussian/CFD/Lagrangian methods can excel; when constrained to sparse, rapid-deployment data (our target use case), they either underperform or become non-operational.

3. The main figures (1, 2, 3, etc.) are referenced but not included in the manuscript text file. Key figures, especially the one illustrating the overall accounting concept, should be integrated into the main text for clarity.

【Reply】 Thank you for noting the problem of figures. We have indeed prepared six figures (with Figure 2 updated), and they were originally embedded in the main text. However, per the editorial instruction in the current revision cycle—”Please remove your figures from within your manuscript file, leaving only the individual TIFF/EPS image files, uploaded separately. These will be automatically included in the reviewers’ PDF.”—we removed in-text embeds and placed the figure callouts and captions in the end-of-manuscript appendix, while uploading all figures as standalone TIFF/EPS files. Our understanding is that the journal’s system will auto-assemble these into the reviewer PDF; if preferred, we can reinsert low-resolution placeholders in the main text for clarity in the next round.

4. The writing is generally good but occasionally overly complex. Some phrases, like "competent verification," are vague. The manuscript would benefit from a thorough proofread to improve clarity and flow.

【Reply】 Thank you for the constructive comment. We have rechecked the whole paper and made revisions to the following phrases:

"competent verification"

Replace with "independent third-party verification".

"near real time"

Replace with "processing latency under 5 minutes".

"accuracy and operational practicality"

Replace with "average absolute error < 10\% and setup time < 2 hours".

"strong plume rise or chemistry dominated cases"

Replace with "cases with significant buoyant plume rise or reactive chemistry (e.g., oxidation, secondary aerosol formation)".

"data-driven time-series learning"

Replace with "LSTM-based next-step regression on trajectories (20 s windows, MSE loss)".

"off-site reverse deduction"

Replace with "off-site inverse accounting".

5. It is unclear how the LSTM-generated virtual paths, which are discrete time series, are directly used in these continuous-time moment formulas. The transition from data to parameters needs a step-by-step explanation.

【Reply】Thank you for this suggestion. We have fully taken this suggestion and added one paragraph before Table 1 of parameter values:

“We take the following steps. (1) Treat the LSTM outputs $\{(\tilde X_{i,t},\tilde Y_{i,t},\tilde Z_{i,t})\}_{i=1,\dots,m;\ t=1,\dots,60}$ as discrete evaluations of the continuous processes at integer seconds; form per-time empirical moments $\bar x(t)=\frac{1}{m}\sum_i \tilde X_{i,t}$, $\bar y(t)=\frac{1}{m}\sum_i \tilde Y_{i,t}$, $q_x(t)=\frac{1}{m}\sum_i \tilde X_{i,t}^2$, $q_y(t)=\frac{1}{m}\sum_i \tilde Y_{i,t}^2$, and $\bar z(t)=\frac{1}{m}\sum_i \tilde Z_{i,t}$ (clip $\tilde Z_{i,t}\leftarrow\max\{\tilde Z_{i,t},0\}$ if needed). (2) Estimate drifts via regression-through-origin implied by $E[H_x(t)]=\mu_x t$, $E[H_y(t)]=\mu_y t$: $\hat\mu_x=\sum_{t=1}^{60} t\,\bar x(t)/\sum_{t=1}^{60} t^2$, $\hat\mu_y=\sum_{t=1}^{60} t\,\bar y(t)/\sum_{t=1}^{60} t^2$. (3) Estimate horizontal volatility from second moments using $E[H_x^2(t)]=\sigma^2 t+(\mu_x t)^2$ and analogously for $y$: $\hat\sigma_x^2=\frac{1}{60}\sum_{t=1}^{60}[q_x(t)-(\hat\mu_x t)^2]/t$, $\hat\sigma_y^2=\frac{1}{60}\sum_{t=1}^{60}[q_y(t)-(\hat\mu_y t)^2]/t$, then $\hat\sigma=\sqrt{\tfrac{1}{2}(\hat\sigma_x^2+\hat\sigma_y^2)}$; estimate vertical volatility from the truncated expectation $E[V(t)]=\sigma_z \sqrt{t}/\sqrt{2\pi}$ via $\hat\sigma_z=\frac{\sqrt{2\pi}}{60}\sum_{t=1}^{60}\bar z(t)/\sqrt{t}$, which matches Eqs.~\eqref{moment1}–\eqref{moment2}. Based on the parameter estimation of $\mu_x$, $\mu_y$, $\sigma$, $\sigma_z$, we present all values of main parameters in Table~\ref{tab:testpara} below.”

Thanks again for your suggestion and comments, which have greatly improved this research article, hopefully our revision can answer your questions and meet the requirements.

**For reviewer 2:**

1. The authors employ Brownian motion to describe carbon molecular diffusion. However, in open atmospheric environments, carbon dispersion is typically governed by turbulent transport rather than molecular-scale random motion. The manuscript should justify why a Geometric Brownian Motion (GBM) framework was chosen to represent atmospheric diffusion and clarify its advantages or approximations relative to conventional plume or turbulence models.

【Reply】 Thank you for raising this important point. We agree that open-atmosphere dispersion is dominated by turbulent transport, our choice of a drifted Brownian/GBM framework is a deliberate coarse-grained approximation that aggregates unresolved turbulent fluctuations into an effective stochastic process. Specifically, mean wind advection is represented by drift, while rapidly decorrelating shear, gusts, and small eddies are lumped into a volatility term, consistent with the diffusion limit of many random impulses and the Maxwell–Boltzmann–inspired Gaussianity of velocity components over short horizons. This surrogate is not intended to replace plume or turbulence-resolving models (Gaussian plume/puff, Lagrangian particles, or CFD), which require detailed, time-resolved meteorology (stability class, PBL height, plume rise), terrain/roughness, and multi-sensor data—inputs that our low-cost, rapid-deployment protocol purposefully avoids. Under these sparse-data conditions, the Brownian formulation offers (i) parsimony and closed-form arrival probabilities for inversion, (ii) local calibratability from brief on-site trajectory snippets via the LSTM, and (iii) operational robustness when full turbulence characterization is infeasible. We clarify these modeling assumptions, delineate the regime of validity (short-horizon, near-field, episodic releases with limited met data), and add sensitivity tests to demonstrate that the inferred emission rates are stable to reasonable variations in the effective volatility and drift, while acknowledging scenarios (complex terrain, strong plume rise, stratified layers) where turbulence-resolving models are preferable. Hence we have added one more paragraph as follows:

“To accurately describe molecular movements, Brownian motion presents a natural stochastic scaffold because it emerges as the macroscopic limit of innumerable microscopic collisions whose velocities follow the Maxwell–Boltzmann distribution (refer to \cite{Shavit}). In a thermally agitated medium, particle velocity components are approximately independent Gaussian variables; successive collisions randomize directions and magnitudes, and, under standard diffusion scaling, the cumulative displacement converges to a (drifted) Brownian process. Our use of a GBM-like representation is thus an effective, coarse-grained surrogate for unresolved turbulence at the small sampling scales of our field protocol: the horizontal drift terms proxy mean wind advection, while the volatility terms encapsulate aggregated, rapidly decorrelating fluctuations from shear, gusts, and micro-scale eddies. We emphasize that this is an approximation, not a replacement for plume or turbulence-resolving models; however, in sparse-data, rapid-deployment settings where stability class, boundary-layer depth, plume rise, and high-resolution meteorology are unavailable, the Brownian framework offers a parsimonious, closed-form route to arrival probabilities and inversion, with parameters that can be locally calibrated from short trajectory snippets (via LSTM) to reflect near-term conditions. This maintains physical grounding through intuition of kinetic-theory while ensuring operational tractability and reproducibility under practical data constraints.”

2. The paper repeatedly highlights that the LSTM “generates virtual diffusion paths,” but does not specify the model’s training objective, input variables, or the mechanism by which the LSTM output is coupled with Brownian motion parameters. A detailed explanation of this hybrid modeling strategy is necessary for the study to be scientifically credible and reproducible.

【Reply】Thank you for the valuable comment. In the revision, we explicitly define the hybrid strategy and why it improves on a Brownian-only approach. The LSTM is trained as a univariate next-step regressor on carbon-trajectory sequences using 20-second sliding windows as inputs and x(t+1) as the target, with mapminmax scaling, MSE loss, Adam (lr=0.01), mini-batch 32, up to 100 epochs, and an 80/20 chronological split for evaluation; multi-step (up to 90 s) forecasts are produced recursively. These LSTM forecasts ‘generate virtual diffusion paths’ that encode short-horizon, potentially nonstationary dynamics. We then couple them to the Brownian framework by estimating drift and volatility parameters from the LSTM-generated sample paths via the moment formulas (Eq. (0.2)–(0.3)), which feed the closed-form arrival probability Ωt and the inversion statistic D(v). This is superior to a direct Brownian fit because Brownian inference is dominated by historical averages (thus biasing drift toward the longer historical segment), whereas the LSTM prioritizes short-term predictiveness. As illustrated in Figure~\ref{fig:lstmgraphs}, when the historical series trends negative, a Brownian-only fit yields a negative drift and ignores the short reversal at the end of the window; the LSTM captures this brief positive turn and yields predictions that connect smoothly to observations (see the red segment in the right panel). Hence, our LSTM–Brownian coupling provides a scientifically transparent, reproducible pipeline: LSTM for short-term trajectory forecasting; moment-based mapping of those forecasts to Brownian parameters; and closed-form diffusion-accounting for emission inversion. Also we have added the above arguments into the Section “Analysis on real case of burning straw”:

“In addition, our hybrid design outperforms a Brownian-only specification because the latter primarily reflects long-horizon historical averages, whereas the LSTM is explicitly optimized for short-term prediction. As illustrated in Figure~\ref{fig:lstmgraphs}, when the historical sequence trends negative, a direct Brownian fit yields a negative drift and effectively discards the short reversal at the end of the window; by contrast, the LSTM captures this brief positive turn and produces forecasts that connect smoothly to the observed trajectory (see the red segment in the right panel). Consequently, we first use the LSTM to generate short-horizon virtual diffusion paths under current conditions, and then map those paths to Brownian drift and volatility via the moment estimators in Eqs. (0.2)–(0.3), ensuring that the stochastic parameters reflect near-term dynamics rather than being anchored solely to the past.”

3. The final section discussing the design of a carbon penalty rate, while practically interesting, is disconnected from the core modeling and empirical analysis of the paper. It should either be removed or significantly condensed, as it introduces speculative content beyond the scope of this technical study.

【Reply】 Thank you for the constructive feedback. We agree that the carbon penalty rate discussion was insufficiently connected to the paper’s core modeling and empirical analysis and could be viewed as a speculative policy extension beyond the technical scope. To improve focus and coherence, we have removed that paragraph in the revised manuscript:

“It may contribute to design a penalty rate for agricultural carbon emission. The penalty rate should be a per-tonne charge applied only to the conservatively estimated excess. Let the billable excess be the lower 95% confidence estimate of the event’s emissions above any permits already surrendered. The cash penalty per tonne is set to 1.2 times the 30-day average carbon price in the relevant ETS, with a hard floor (e.g., 100EUR/tCO2e in the EU or a policy floor in China). If the emitter self-reports within 48 hours and cooperates with mitigation, the rate is discounted by 25%; if not self-reported, it is surcharged by 25%; repeat events within 12 months add 0.1 to the multiplier. This keeps penalties market-consistent, discourages concealment, and respects the measurement uncertainty inherent to our method.”

In the new version, the conclusion remains narrowly focused on the methodological contributions, empirical validation, and clearly bounded future technical work.

4. Although the references cover a broad range of thermodynamic and diffusion studies, most are dated or only tangentially related to carbon accounting. The authors are encouraged to include recent advances in remote sensing–based carbon flux inversion and machine learning approaches for agricultural carbon monitoring to better situate their contribution in the current research landscape.

【Reply】 Thank you for this helpful suggestion. We agree that our initial references leaned toward classical thermodynamics and diffusion literature and did not sufficiently reflect recent advances in remote sensing–based carbon flux inversion and machine learning for agricultural carbon monitoring. To better situate our contribution within the current landscape, we have updated the Related Work/Literature Review to foreground state-of-the-art methods that span policy inventories, near-field inversion, stochastic transport, high-fidelity CFD with ML augmentation, satellite top-down flux estimation and downscaling, and compliance-grade stack monitoring. These additions emphasize recent progress in multi-source data fusion, hybrid training, and scalable, real-time monitoring frameworks relevant to agricultural contexts and rapid-deployment scenarios.

Concretely, we have added the following integrative paragraph to position our work among contemporary methods with 7 additional references: “Contemporary carbon monitoring blends complementary methods across scales and costs. First, activity-based emission factors remain foundational for policy inventories and sector benchmarking, with recent work emphasizing nationally localized strategies and evolving effective factors under climate and management change \cite{cmc1,cmc2}. For near-field dispersion from point or area sources, Gaussian plume/puff analytics deliver fast forward models suited to inversion with time-varying winds and sparse sensors \cite{cmc3}. To better capture unsteady transport and intermittency, Lagrangian particle models synthesize realistic single-particle trajectories and extreme events for stochastic reconstruction and data augmentation \cite{cmc4}. Where site complexity or building wakes matter, high-fidelity CFD (RANS/LES) is increasingly augmented by machine learning for accelerated solvers, improved closures, and reduced-order surrogates \cite{cmc5}. At regional-to-national scales, satellite top-down approaches (for example, using NO2/CO proxies and hybrid learning) enable daily 10-km downscaling of CO2 fluxes to verify mitigation at city and county levels \cite{cmc6}. Finally, on-site CEMS provides hourly, facility-level stack measurements that quantify compliance and reveal the impacts of strengthened standards and ultra-low-emission retrofits in heavy industry \cite{cmc7}. In the sequel, we summarize the above-mentioned methodologies, their key characteristics are compared in Table~\ref{ref:summary} below.”

Thanks again for your suggestion and comments, which have greatly improved this research article, hopefully our revision can answer your questions and meet the requirements.

**For Additional Editor Comments:**

1. Further revisions are still needed regarding technical clarity, novelty, and experimental verification.

【Reply】 Thank you for the constructive summary. We have revised the manuscript to improve technical clarity, highlight novelty, and strengthen experimental verification. Specifically, at the end of the Introduction we added a concise “Highlights and novelties” paragraph to explicitly articulate our methodological contributions: “Highlights and novelties of this research include several significant contributions. (1) This paper transcends the limitations of conventional carbon accounting by eliminating dependency on carbon emission factor methodologies while simultaneously addressing monitoring challenges that CEMS approaches cannot resolve, successfully implementing an ‘off-site inverse accounting’ carbon monitoring methodology specifically calibrated for agricultural contexts. (2) It pioneers the integration of LSTM neural networks with traditional Geometric Brownian Motion modeling, enabling streamlined parameter updating based on actual on-site meteorological measurements, thereby enhancing both accuracy and operational efficiency.” We also clarified how the inverse framework operates under sparse, rapidly changing field conditions and how the LSTM-calibrated parameters map to the stochastic dispersion model used for off-site inference.

To reinforce experimental verification, we expanded the case-study section by detailing the field protocol, parameter-estimation pipeline, and robustness checks (multiple sensor locations and time-window variations), and by reporting the resulting uncertainty bounds. We added cross-method comparisons (e.g., plume-style baselines where appropriate) and reported consistency across repeated trials to demonstrate reproducibility. Together, these revisions improve transparency of the algorithmic steps, foreground the novelty relative to emission-factor and on-site CEMS approaches, and provide more comprehensive evidence that the proposed method achieves accurate, operationally feasible accounting in agricultural scenarios.

Thanks again for your suggestion and comments, which have greatly improved this research article, hopefully our revision can answer your questions and meet the requirements.
